# Celastrol Induces Autophagy by Targeting AR/miR-101 in Prostate Cancer Cells

**DOI:** 10.1371/journal.pone.0140745

**Published:** 2015-10-16

**Authors:** Jianquan Guo, Xuemei Huang, Hui Wang, Huanjie Yang

**Affiliations:** School of Life Science and Technology, Harbin Institute of Technology, Harbin, 150001, China; Innsbruck Medical University, AUSTRIA

## Abstract

Autophagy is an evolutionarily conserved process responsible for the degradation and recycling of cytoplasmic components through autolysosomes. Targeting AR axis is a standard strategy for prostate cancer treatment; however, the role of AR in autophagic processes is still not fully understood. In the present study, we found that AR played a negative role in AR degrader celastrol-induced autophagy. Knockdown of AR in AR-positive prostate cancer cells resulted in enhanced autophagy. Ectopic expression of AR in AR-negative prostate cancer cells, or gain of function of the AR signaling in AR-positive cells, led to suppression of autophagy. Since miR-101 is an inhibitor of autophagy and its expression was decreased along with AR in the process of celastrol-induced autophagy, we hypothesize that AR inhibits autophagy through transactivation of *miR-101*. AR binding site was defined in the upstream of *miR-101* gene by luciferase reporter and ChIP assays. MiR-101 expression correlated with AR status in prostate cancer cell lines. The inhibition of celastrol-induced autophagy by AR was compromised by blocking miR-101; while transfection of miR-101 led to inhibition of celastrol-induced autophagy in spite of AR depletion. Furthermore, mutagenesis of the AR binding site in *miR-101* gene led to decreased suppression of autophagy by AR. Finally, autophagy inhibition by miR-101 mimic was found to enhance the cytotoxic effect of celastrol in prostate cancer cells. Our results demonstrate that AR inhibits autophagy *via* transactivation of *miR-101*, thus combination of miR-101 mimics with celastrol may represent a promising therapeutic approach for treating prostate cancer.

## Introduction

Autophagy is a conserved process responsible for the turnover of long-lived proteins or removal of damaged organelles in eukaryotic cells. It is regulated by series of autophagy related genes (ATGs) that are involved in the initiation, autophagosome formation and maturation [1]. Autophagy normally occurs at low basal levels, but is induced in response to stress stimulations, including starvation, hypoxia, or hormone deprivation [2]. Increased autophagy may provide a benefit for cancer cells to deal with the conditions that are not favorable to survive.

Celastrol, a triterpenoid isolated from the traditional Chinese medicine ‘Thunder of God Vine’ has shown effectiveness against human prostate cancer *in vitro* and *in vivo* [3, 4]. It promotes destabilization of androgen receptor (AR) through inhibition of Hsp90 or activation of calpain [5, 6]. AR is a member of the steroid superfamily of ligand activated transcription factors. It plays an important role in the development and progression of prostate cancer, thus androgen deprivation therapy through medical or surgical castration is a standard strategy for the treatment of prostate cancer [7]. Blocking AR signaling pathway has been shown to trigger autophagy in AR positive prostate cancer cell lines, which is favorable for cell survival or cell death depending on the applied specific inhibitors and the cell contexts [8–11]. Induction of autophagy was found to improve cell viability upon androgen deprivation and hypoxia or under starvation conditions [8, 9, 11]. AR degrader was shown to induce cell death via induction of autophagy [10]. Several molecules, such as Grp78 and AMPK have been demonstrated to be involved in the regulation of androgen deprivation induced autophagy [8, 9]. However, as a transcription factor, the mechanism by which AR regulates autophagy has not been fully understood. Our microarray data showed that miR-101 expression was down-regulated when autophagy was induced by celastrol. MiR-101 has been reported as an inhibitor of autophagy, which suppresses both induction and maturation of autophagy by targeting *ATG4D*, *RAB5A* and *STMN1* [12]. In addition, an AR binding site was predicted in the upstream region of the *miR-101* gene [13]. These findings prompted our hypothesis that celastrol induces autophagy by targeting AR/miR-101 in prostate cancer cells. In the present study, we verified the AR binding site in the upstream region of the *miR-101* gene by luciferase reporter and ChIP assays. The expression of miR-101 was found to correlate with AR status in prostate cancer cell lines. Despite AR depletion by celastrol, transfection of miR-101 mimic in LNCaP cells led to inhibition of autophagy. When miR-101 was blocked, AR inhibition on autophagy was relieved. Furthermore, mutagenesis of the AR binding site in the upstream region of the *miR-101* gene led to decreased inhibition of AR-mediated autophagy.

## Materials and Methods

### Chemical and oligonucleotides

Celastrol was purchased from Cayman chemical company (Ann Arbor, MI, USA). All the oligonucleotides were synthesized by Ribio (Guangzhou, China) with the following sequences: miR-101 mimic, 5'-UACAGUACUGUGAUAACUGAA-3'; miRNA mimic Negative control (Ncontrol) #22: 5'-UUUGUACUACACAAAAGU ACUG-3', miR-101 inhibitor: 5'-UUCAGUUAUCACAGUACUGUA-3'; miRNA inhibitor Ncontrol #22: 5'-UCACAACCUCCUAGAAAGAGUAGA-3'. siRNA for AR: 5'-GGTGATCACAGGATAGGTATT-3', siRNA for control: 5'-GGGCCATGGCA CGTACGGCAAG-3'.

### Cell culture and cell transfection

Human prostate cancer cell lines LNCaP, 22Rv1, DU145 and PC-3 were obtained from Shanghai Institute of Biochemistry and Cell Biology (Shanghai, China), and cultured in RPMI 1640 (Gibco BRL Co. Ltd., USA) supplemented with 10% fetal bovine serum (Biological industries, Israel) and 1% penicillin/streptomycin in a 37°C humidified atmosphere containing 5% CO_2_/95% air.

For androgen starvation, LNCaP cells were cultured in phenol red-free RPMI 1640 media (Gibco BRL Co. Ltd., USA) containing 1% charcoal-stripped FBS (Invitrogen, Eugene, OR, USA) for 24 h before experiments.

Transfection was performed by using Lipofectamine 2000 (Invitrogen, Eugene, OR, USA). Transient transfection of pEGFP-C1-AR or empty vector (Addgene) was performed in LNCaP or PC-3 cells. After transfection, medium were replaced with fresh medium containing 1 nM of R1881. Stable transfection of pEGFP-C1-AR or empty vector was performed in DU145 cells. Cells were pretreated with R1881 (1 nM) for 24 h before experiment. LNCaP cells were transfected with pEGFP-LC3 (Addgene) and selected with 0.8 mg/ml G418 (Calbiochem, Merck KGaA, Darmstadt, Germany) to obtain stable transfectants.

### Plasmids

pGL3-Basic was a generous gift from Dr. Li Yu (Harbin Institute of Technology, China). To generate reporter constructs, pGL3-B-miR-101-L and pGL3-B-miR-101-S, a 1793 bp DNA fragment in the upstream of *miR-101* gene that contains the predicted AR binding site and its shorter fragment with deletion of the predicted AR binding site were amplified using primers 5'-CGCACGCGTAATGGATTTATTTCCTACCCT ACAT-3'; 5'-CCGCTCGAGTATTCCCTGCCACCCAGCTCACC-3', and 5'-CGCAC GCGTAATGGATTTATTTCCTACCCTACAT-3'; 5'-CCGCTCGAGTATTCCCTGCC ACCCAGCTCACC-3', respectively, and cloned into pGL3-Basic vector. The reporter construct that contains a 300 bp fragment with the predicted wild-type AR binding site, pGL3-B-miR-101-WBS, was amplified with PCR using primers 5'-CGCACGCGTAATGGATTTATTTCCTACCCTACAT-3', and 5'-CCGCTCGAG CTTTCTTCTTTTGTTTTTCATTTTC-3'. To mutagenize the predicted AR binding site in pGL3-B-miR-101-WBS (designated pGL3-B-miR-101-MBS), two-step PCR was performed. For the first step PCR, the following primers (mutated nucleotides were indicated by lower cases): 5'-CGCACGCGTAATGGATTTATTTCCTACCC TACAT-3'; 5'-TAACTTTAtAgaATATTtaAgATAG-3', 5'-CTATcTtaAATATtcTaTAA AGTTA-3'; and 5'-CCGCTCGAGCTTTCTTCTTTTGTTTTTCATTTTC-3' were used. The PCR products were used as the template for the second step PCR using the outer pair of primers.

PGL3-B-miR-101W construct was generated by PCR amplification of a 2166 bp DNA fragment containing both AR binding site and miR-101 gene sequence using primers 5'-CGCACGCGTGCCTTGGTCAGACTGGAT-3', 5'-CCGCTCGAGAT GTTACCACGCCATTTA-3', and cloning into the pGL3-Basic vector. Its mutant form of construct, PGL3-miR-101M, was generated with two-step PCR as described above using two pairs of primers containing a mutant AR binding site 5'-CGCACGCGTGCCTTGGTCAGACTGGAT-3'; 5'-TAACTTTAtAgaATATTtaAgATAG-3', and 5'-CTATcTtaAATATtcTaTAAAGTTA-3'; 5'-CCGCTCGAGATGTT ACCACGCCATTTA-3'. Letters in lower case indicate the mutated nucleotides.

### Quantitative real-time PCR

Total RNA was extracted from cells using TRIzol reagent (Invitrogen, Eugene, OR, USA). A total of 1.0 μg of total RNA was used to synthesize complementary DNA using EasyScript First-Strand cDNA Synthesis SuperMix (Transgen, Beijing, China). qPCR was performed to determine the expression levels of each gene using the following primers: ATG5: 5'-GCAAGCCAGACAGGAAAAAG-3', 5'-GACCTTC AGTTGGTCCGGTAA-3'; ATG7: 5'-CAGGAGATTCAACCAGAGAC-3', 5'-AGAT ACCATCAATTCCACG G-3'; AR: 5'-CAGCAACAGCAGCAGGAAGC-3'; 5'-CTTT TGCATTCGGCCAATGG-3'; GAPDH: 5'-TGCACCACCAACTGCTTAGC-3'; 5'-G GCATGGACTGTGGTCATGAG-3'. Pri-miR-101: 5'-GTATTTCGTAGGACAGG-3'; 5'-TCTACAGGAAGCGAGT-3'. Data was normalized to GAPDH expression levels.

MiRNA expression was analyzed as reported by Shi *et al* [14]. Briefly, total RNA (1 μg) was polyadenylated using Poly(A) Tailing Kit (Ambion Inc, Foster, CA) following the manufacturer’s instruction. After phenol-chloroform extraction and ethanol precipitation, the RNA was reverse-transcribed with EasyScript reverse transcriptase (Transgen, Beijing, China) and 0.5 μg of poly(T) adapter 5'-GCTGTCA ACGATACGCTACGTAACGGCATGACAGTGT(24)N(A/C/G)-3' according to the manufacturer’s protocols (Invitrogen, Eugene, OR, USA). MiR-101 expression was determined using primers 5'-GCTGTCAACGATACGCTA-3' and 5'-CAGTACTGTG ATAACTGAA-3'. The data was presented as an average of three experiments and normalized to the expression of endogenous U6 RNA using ΔΔCT method.

### Western blot analysis

Whole cell lysates were prepared using RIPA buffer containing 10 mM Tris-HCl (pH 8.0), 150 mM NaCl, 0.1% sodium dodecyl sulfate, 0.8% Triton X-100 and protease inhibitor cocktail (Roche, Mannheim, Germany). Western blotting was performed as described in [15]. Briefly, 40 to 100 μg proteins per well were separated by polyacrylamide gel and then electro-transferred to PVDF membranes. After 2 h of blocking, the membranes were washed and probed with the following primary antibodies: rabbit anti-LC3, mouse anti-p62 and anti-PARP (Cell Signaling Technology, USA), mouse anti-GAPDH (KC-5G4) (KANGCHEN, shanghai, China), mouse AR (sc-816) and mouse PSA (sc-7316) (Santa Cruz Biotechnology, CA, USA). Protein expression was detected by ECL (PPLYGEN, Beijing, China) and visualized using LI-COR Odyssey 2800 (LI-COR Biosciences, Lincoln, NE, USA).

### Luciferase reporter assays

DU145 prostate cancer cells were plated in 24-well plates (1×10^5^ per well) for 24 h and co-transfected with 300 ng of reporter constructs (pGL3-B-miR-101-L, pGL3-B-miR-101-S, pGL3-B-miR-101-WBS, pGL3-B-miR-101-MBS or pGL3-Basic), 300 ng of pEGFP-C1-AR or pEGFP-C1 plasmids and 10 ng of pRLSV40 (Promega, San Luis Obispo, CA, USA) using Lipofectamine 2000. After transfection, the medium was replaced with fresh medium containing R1881 (1 nM) and harvested 24 h later. Luciferase activities were assayed using the dual-luciferase reporter assay system (Promega, San Luis Obispo, CA, USA) in a Turner TD20/20 luminometer and normalized to Renilla luciferase activity.

### ChIP assays

ChIP was performed as previously reported [16]. Briefly, DU145 or LNCaP cells were transfected with pEGFP-C1-AR or pEGFP-C1. After 24 h transfection, medium was replaced with fresh medium containing 1 nM of R1881 overnight. Cells were harvested and cross-linked with 1% formaldehyde for 10 min at 37°C. Fixed cells were washed with ice-cold phosphate-buffered saline, and resuspended in ice-cold hypotonic buffer (10 mM HEPES, 1.5 mM MgCl_2_ and 10 mM KCl), followed by homogenization. Collected nuclei were resuspended in hypotonic buffer and sonicated to an average DNA length of 200 to 1000 bp. Aliquots (1%) of sheared DNA were removed as input, and the remainder was used for individual ChIP reaction equally. The chromatin solutions were precleared by addition of protein A-sepharose (GE Healthcare, Fairfield, USA) for 2 h and incubated with 10 μg of AR antibody or IgG as a negative control overnight at 4°C. The protein/DNA immune complexes were collected by centrifugation at 4°C, washed with dilution buffer (20 mM Tris, 150 mM NaCl, 1% Triton X-100, 2 mM EDTA and protein inhibitor cocktail), and elution buffer (0.1M NaHCO_3_ and 1% SDS). Protein/DNA cross-links were reversed by addition of NaCl to a final concentration of 200 mM followed by incubation at 65°C for 4 hr. After DNA purification, PCR was performed to detect miR-101 expression using the following primers: 5'-CGCACGCGTAATGGATTTATTTCCTACCCTA CAT-3', and 5'-CCGCTCGAGCTTTCTTCTTTTGTTTTTCATTTTC-3'. The PCR products were analyzed on agarose gels (2%) with ethidium bromide.

### Confocal microscopy

LNCaP cells with GFP-LC3 expression were seeded in 6-well plates. After celastrol treatment, the cells were fixed in 4% paraformaldehyde for 15 min and washed with PBS three times. Cells were then permeabilized with 0.05% (v/v) of Triton X-100, stained with DAPI (Invitrogen, Eugene, OR, USA) and analyzed using confocal microscope (ZEISS, LSM510). The number of GFP-LC3 punctate was quantified. Positive cells which contained five or more puncta were selected. Fifty cells per condition per experiment were analyzed.

### MTT assays

LNCaP cells were plated in a 96-well plate (5000/well). After treatments, MTT (5 mg/ml) was added and incubated at 37°C for 4 h to allow for complete cleavage of the tetrazolium salt by metabolically active cells. Cell lysis buffer (20% SDS, 20 mM HCl) was added to each well, followed by colorimetric analysis using an iMark Microplate Absorbance Reader (Bio-Rad, Hercules, CA, USA) at 595 nm.

### Colony formation assays

LNCaP cells were treated with celastrol in the presence or absence of miR-101 mimic or negative control or bafilomycin A1 (Sangon biotech, shanghai, China) for 24 h. After treatments, LNCaP cells (500/well) were seeded in six-well plate and cultured for 15 days without disturbance. Cells were then fixed with 4% formaldehyde in PBS and stained with crystal violet. Colonies with over 50 cells were counted.

### Statistic analysis

Statistical analysis was performed using SPSS statistical software. All values were expressed as the mean ± SD, two group comparisons were carried out using paired Student’s t-test. Data from multiple groups was analyzed by one-way ANOVA, followed by Dunnett's test. For all the tests, the level of significance was defined as * or #, *P*<0.05; **, *P*<0.01.

## Results

### Celastrol induces autophagy in human prostate cancer cells

**Fig 1:**
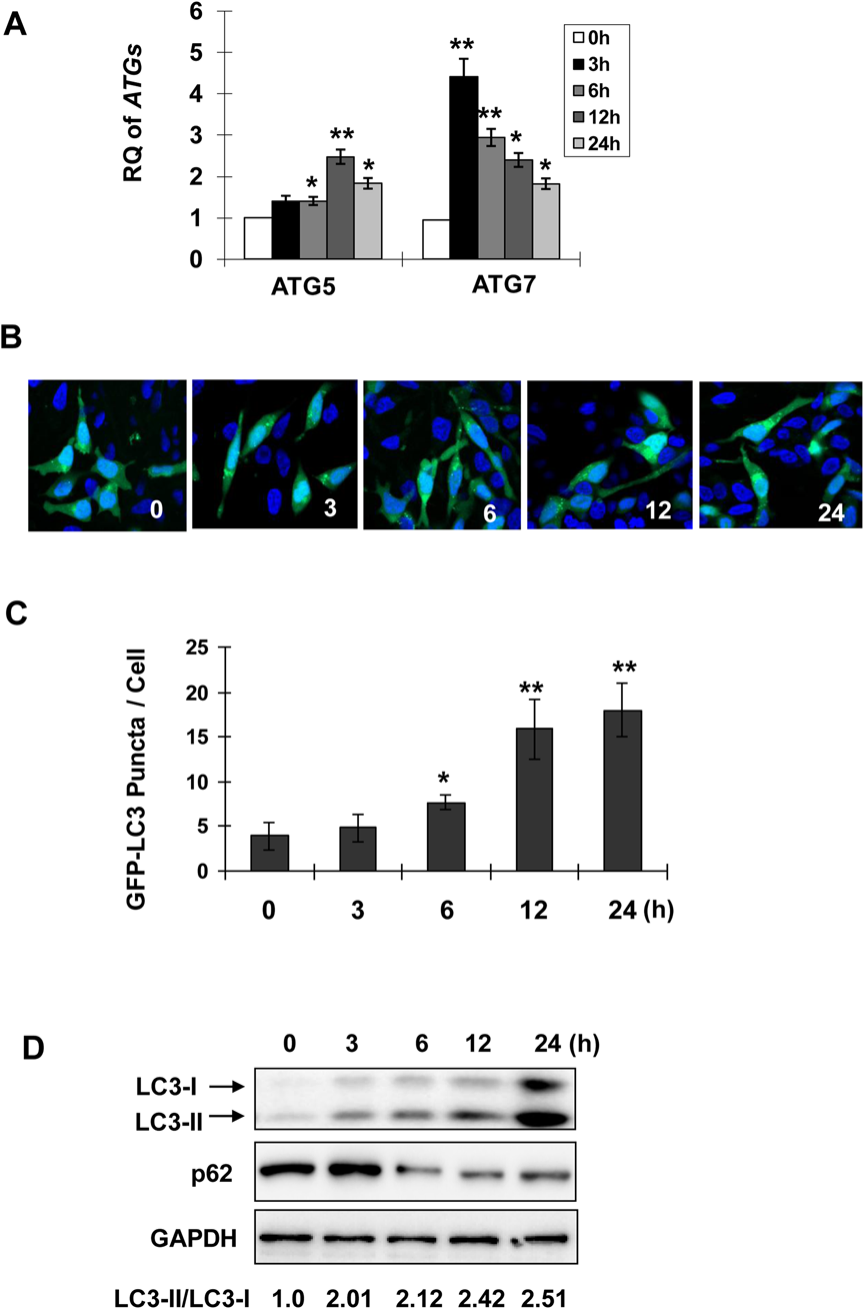
Celastrol triggers autophagy in prostate cancer cells. Parental LNCaP cells (**A**, **D**) or the cells transfected with GFP-LC3 (**B**, **C**) were treated with celastrol at 2.0 μM for indicated times. mRNA expressions of autophagy related genes were measured by qPCR (**A**). RQ, relative quantity. GFP-LC3 transfected cells were stained by DAPI after treatments. GFP-LC3 puncta were observed under confocal microscope (**B**) and quantified in **C**. The cells that contained over 5 puncta were selected and fifty cells were analysed for each treatment. **D**, Protein extracts were immunoblotted with antibodies against LC3, p62 and GAPDH (loading control). Asterisks denote significance compared with control (0 h). *, *P* <0.05; **, *P* <0.01.

Targeting AR with celastrol for prostate cancer treatment has been shown by several groups [3, 5]. Since AR inhibition is associated with autophagy induction, whether celastrol could induce autophagy in human prostate cancer cells was determined. As shown in Fig 1, induction of autophagy-related genes *ATG5* and *ATG7* was observed at early time points after celastrol treatment in LNCaP prostate cancer cells (Fig 1A). ATG7 acts as the ubiquitin E1-like enzyme while ATG5 acts as the ubiquitination substrate or an E3-like enzyme in the two step ubiquitination process [1]. In accordance with their functions in autophagy induction, *ATG7* showed higher expression and earlier response than *ATG5* upon celastrol treatment (Fig 1A). Both ATG5 and ATG7 are required for the recruitment of LC3, the microtubule-associated protein which is diffused in the cytoplasm, to autophagosome membrane. To visualize LC3 recruitment, LNCaP cells were transfected with a plasmid encoding GFP-tagged LC3. GFP-LC3 puncta were observed to increase significantly after 6 h treatment with celastrol (Fig 1B and 1C). In accordance, conversion from LC3-I to its lipid form LC3-II, the hallmark of autophagy, was detected after 3–6 h treatments and increased noticeably after 12–24 h treatment (Fig 1D). p62 is a receptor on the autophagosome membrane, which is degraded in the lysosome after autophagosome fusion with it [17]. p62 protein level was reduced after 6–24 h treatment with celastrol (Fig 1D), further confirming that celastrol induced autophagy in LNCaP cells.

### AR expression levels inversely correlate with celastrol-induced autophagy

**Fig 2:**
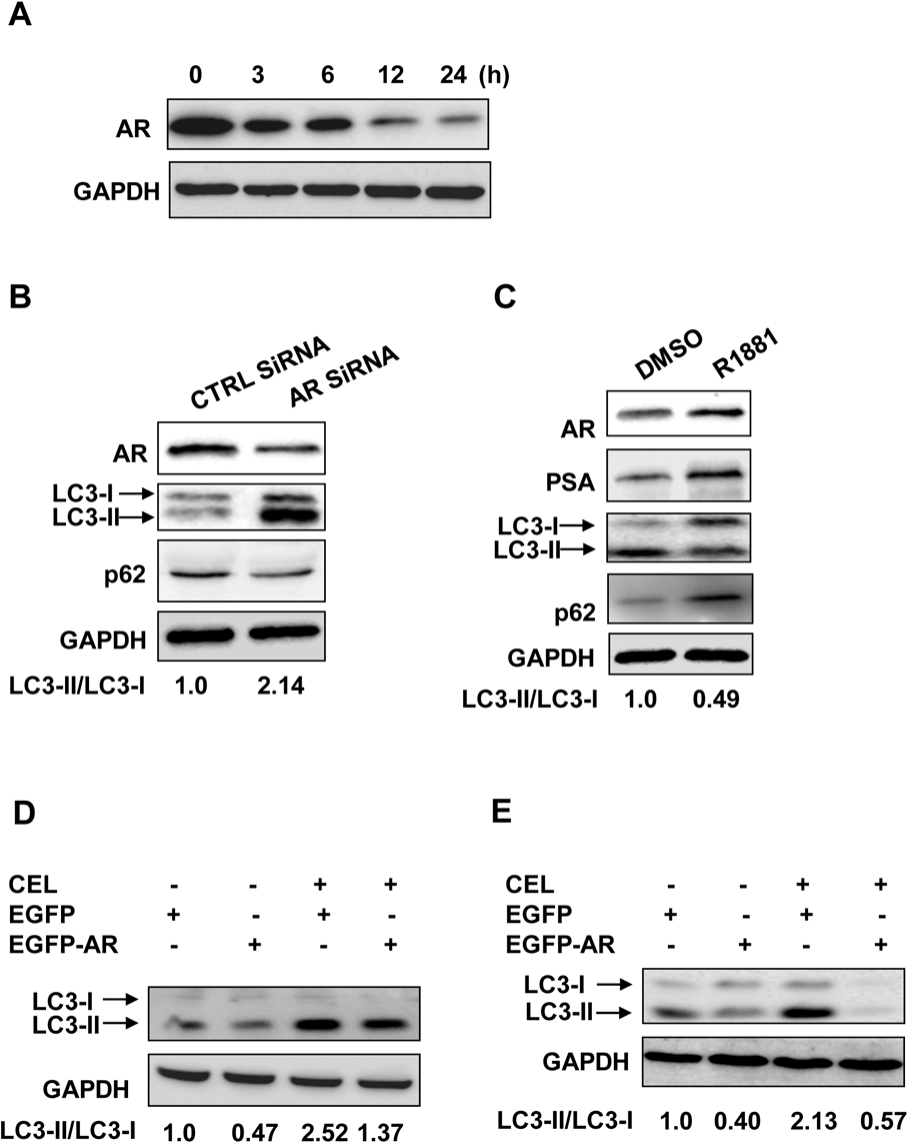
AR suppression on celastrol induced autophagy. **A**, LNCaP cells were treated with celastrol at 2.0 μM for indicated times, the protein level of AR was revealed by Western blotting using GAPDH as a loading control. **B**, LNCaP cells were transfected with AR siRNA or control siRNA for 24 h, the effects on AR knockdown and autophagy induction were verified by Western blotting using AR, LC3, p62 and GAPDH (loading control) antibodies. **C**, LNCaP cells were subjected to androgen starvation as described in "**Materials and Methods**", followed by 1 nM of R1881 treatment for 24 h. AR and its target PSA, as well as autophagic makers LC3 and p62 were detected by Western blotting using GAPDH as a loading control. LNCaP (**D**) or DU145 (**E**) cells were transfected with pEGFP-C1-AR (EGFP-AR) or empty vector (EGFP-V). **D**, After transfection, LNCaP cells were cultured in the medium containing R1881 (1 nM) and treated with or without celastrol (CEL) at 2.0 μM for 24 h (**D**). **E**, DU145 stably transfected cells were pretreated with R1881 (1 nM) for 24 h before celastrol treatment as **D**. LC3 was detected by Western blotting using GAPDH as a loading control.

In the same samples from above, AR protein was decreased by celastrol during autophagy inducing processes. Celastrol treatment caused a substantial decrease in AR expression levels at 3–6 h time points and an almost complete depletion at 12–24 h time points (Fig 2A). In line with the above findings, siRNA knockdown of AR in AR positive LNCaP cells led to ~2-fold increase of LC3-I to LC3-II conversion (Fig 2B). Compared with the control, p62 protein was decreased by AR siRNA (Fig 2B), indicating that AR negatively regulated autophagy. Synthetic androgen R1881 was used to activate endogenous AR signaling in LNCaP cells, which was evidenced by the increased protein levels of AR and its target PSA (Fig 2C). With AR signaling activation, autophagy was inhibited as shown by the increase of p62 level and half-fold decrease of LC3-II/LC3-I ratio (Fig 2C), further confirming that AR plays a negative role in autophagy regulation. In addition, pEGFP-AR was transfected into LNCaP cells. After celastrol treatment, decreased conversion of LC3-I to LC3-II was detected in pEGFP-AR transfected cells compared with the mock transfection (Fig 2D), indicating that forced AR overexpression inhibited autophagy triggered by celastrol. Celastrol also induced autophagy in AR negative DU145 cells, which could be suppressed by exogenous AR (Fig 2E).

### AR mediates the suppression of miR-101 expression by celastrol treatments

**Fig 3:**
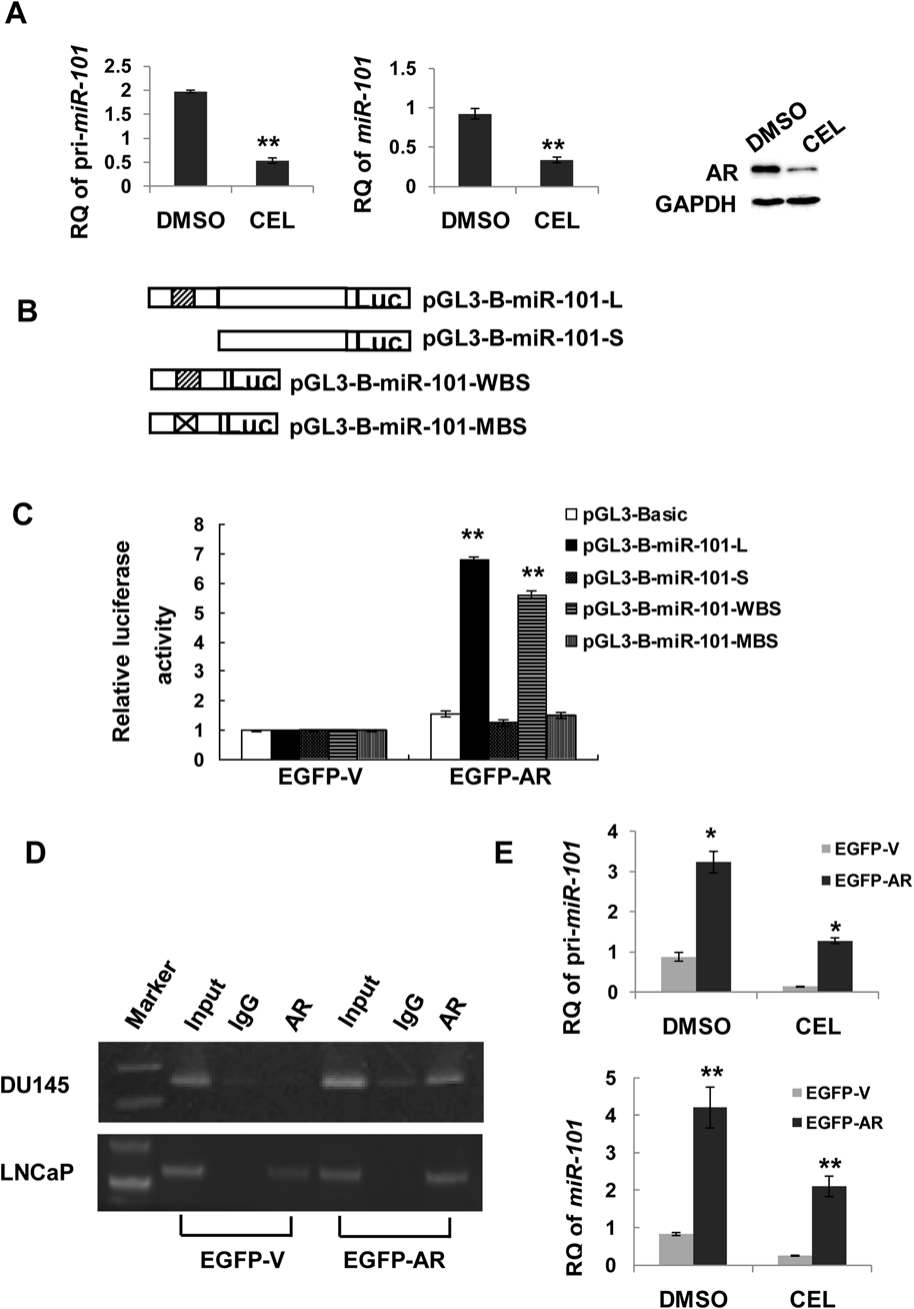
AR suppression mediates miR-101 reduction after celastrol treatment. **A**, miR-101 and AR expressions after celastrol (CEL) treatment. **, *P*<0.01, compared with DMSO. **B**, Schematic depiction of luciferase reporter constructs as detailed in " **Materials and Methods**". Wild type and mutant AR binding sites were indicated by italic dash and cross, respectively. **C**, DU145 cells were transfected with indicated reporter constructs in the presence of pEGFP-C1-AR (EGFP-AR) or pEGFP-C1 (EGFP-V) plasmid for 24 h, and then luciferase activity was detected. pRLSV40 plasmid was co-transfected for normalization. **, *P*<0.01, between EGFP-AR and EGFP-V transfections. **D**, ChIP assay. Cell extracts from DU145 or LNCaP cells transfected with pEGFP-C1-AR (EGFP-AR) or pEGFP-C1 (EGFP-V) were immunoprecipitated with AR antibody or normal mouse IgG. Input was 1/100 of the sonicated chromatin prior to immunoprecipitation. PCR was performed as described in the "**Materials and Methods**". **E**, LNCaP cells transfected with pEGFP-C1-AR (EGFP-AR) or empty vector (EGFP-V) were treated with celastrol (CEL, 2.0 μM) or DMSO for 3 h, pri-miR-101 and mature miR-101 levels were determined by qPCR. Asterisks denote significance between EGFP-AR and EGFP-V transfections. *, *P* <0.05; **, *P* <0.01.

To see miRNA involvement in the process of celastrol-induced autophagy, LNCaP cells were treated with celastrol for 12 h to induce autophagy. MiRNA array was performed and showed that the expression of miR-101, an autophagy inhibitor, was decreased by celastrol (data not shown). Suppression of miR-101 expression by celastrol was confirmed by RT-PCR, which revealed a ~3-fold decrease of pri-miR-101 and mature miR-101 in LNCaP cells post celastrol treatment (Fig 3A, left and middle). This was accompanied by an almost complete depletion of AR (Fig 3A, right), suggesting a positive correlation between AR and miR-101 expression after celastrol treatment. AR is a transcriptional factor and its binding site has been predicted in the upstream region of the *miR-101* gene [13]. Thus, it is conceivable that AR is the mediator of the suppression of miR-101 expression by celastrol. To test this possibility, we first determined if AR could bind to the predicted AR binding site in the upstream region of the *miR-101* gene by luciferase reporter assays. Luciferase reporter constructs were generated as shown in Fig 3B. pGL3-B-miR-101-S (without the predicted AR binding site) showed about 5-fold decreased luciferase activity in comparison with pGL3-B-miR-101-L (with fragments encompassing the predicted AR binding site) (Fig 3C). In addition, pGL3-B-miR-101-MBS in which the AR binding site was mutated showed ~5-fold decreased luciferase activity compared with pGL3-B-miR-101-WBS that contained the wild type binding site (Fig 3C), indicating that this specific sequence is responsible for AR identification. Next, we used ChIP assays to determine if AR could bind to this sequence. DU145 cells were transfected with pEGFP-AR or pEGFP-V. Nuclear proteins were isolated and immunoprecipitated with either AR antibody or control mouse IgG. As expected, miR-101 fragment containing the binding site was specifically amplified in cells transfected with pEGFP-AR (Fig 3D upper), demonstrating that AR could be recruited to this site. In LNCaP cells, *miR-101* fragment was amplified after mock transfection (Fig 3D bottom), showing that endogenous AR could be recruited to the ARE of *miR-101*. Finally, AR overexpression completely rescued pri-miR-101 and mature miR-101 expressions after celastrol treatment, showing AR mediating *miR-101* transcription reduction upon celastrol treatment (Fig 3E).

### AR regulates miR-101 expression in human prostate cancer cells

**Fig 4:**
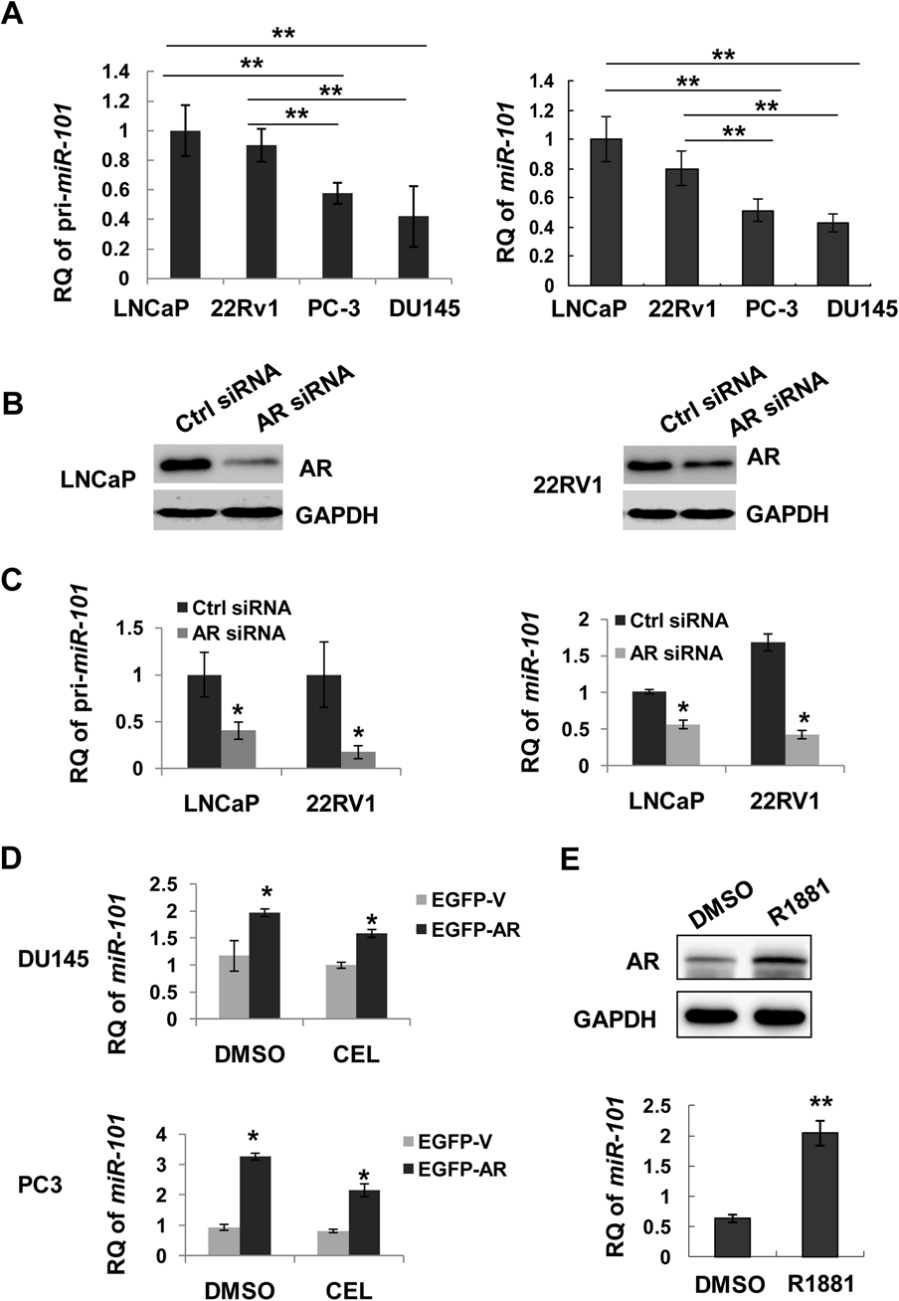
miR-101 expression in prostate cancer cells. **A**, Expressions of pri-miR-101 and mature miR-101 were determined in AR positive or negative cell lines by qPCR. **, *P*<0.01 between two compared groups. **B**, LNCaP or 22Rv1 cells were transfected with AR siRNA or control siRNA (ctrl siRNA). AR knockdown effects were verified by Western blotting. Expressions of pri-miR-101 and mature miR-101 were determined by qPCR (**C).** *, *P*<0.05 *versus* control siRNA. **D**, DU145 or PC-3 cells were transfected with pEGFP-C1-AR (EGFP-AR) or empty vector (EGFP-V) and treated with DMSO or celastrol (CEL, 2 μM) for 24 h. Expressions of mature miR-101 were determined by qPCR. *, *P*<0.05 between EGFP-AR and EGFP-V transfections. **E**, LNCaP cells were treated with R1881 (1 nM) for 24 h after androgen starvation, as described in Fig 2 **C**. AR protein levels were determined by Western blotting using GAPDH as a loading control. MiR-101 expressions were determined by qPCR. **, p<0.01 *versus* DMSO.

AR status could affect miR-101 expression in human prostate cancer cells. Real-Time PCR revealed that the expression levels of pri-miR-101 as well as mature miR-101 were significantly higher in AR-positive LNCaP and 22Rv1 cells than that in AR-negative DU145 and PC3 cells (Fig 4A). AR was knocked down by siRNA in LNCaP (Fig 4B, left) or 22Rv1 cells (Fig 4B, right). AR knockdown resulted in decreased expressions of mature miR-101, which were comparable to the decrease of pri-miR-101 in both LNCaP and 22Rv1 cells (Fig 4C). Forced AR expression caused miR-101 levels increased in AR-negative DU145 and PC3 cells (Fig 4D). Similarly, exogenous AR also increased miR-101 expressions after celastrol treatment (Fig 4D). In LNCaP cells, endogenous AR was re-activated by R1881 after androgen deprivation. With AR activation (Fig 4E, upper), the expression levels of miR-101 were upregulated (Fig 4E). These results demonstrate that AR mainly regulates *miR-101* transcription, but not maturation.

### AR inhibits celastrol-induced autophagy via regulation of *miR-101* in prostate cancer cells

**Fig 5:**
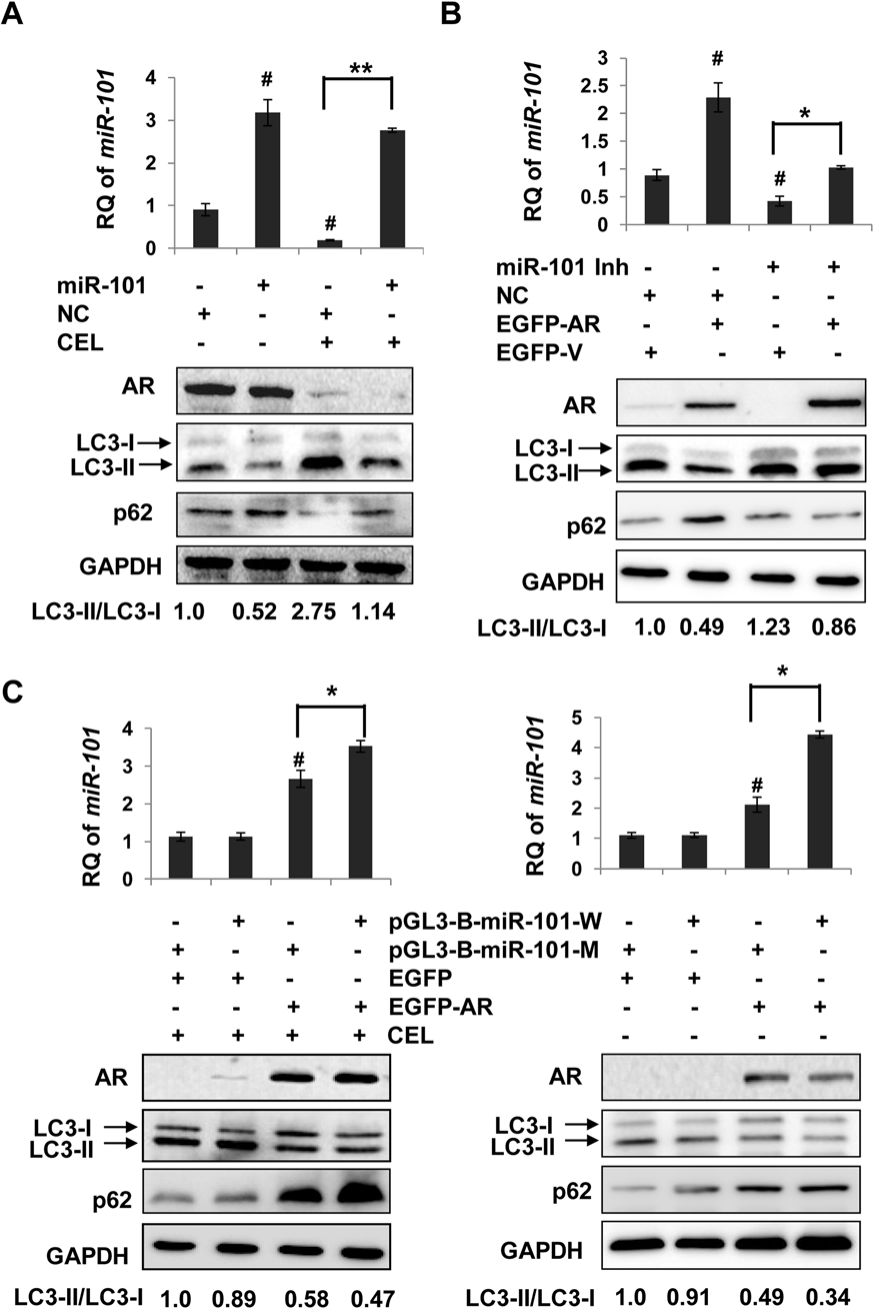
AR inhibition on celastrol-induced autophagy is related with miR-101 transactivation. To see whether miR-101 could affect AR suppression on celastrol-induced autophagy, AR positive LNCaP cells were transfected with miR-101 mimic or negative control (NC) for 24 h (**A**), followed by additional 24 h treatment with celastrol (2.0 μM, CEL). MiR-101 levels were determined by RT-PCR. #, *P*<0.05 *versus* NC transfection without celastrol treament. **, *P*<0.01 between miR-101 and NC transfections with celastrol treatment. The protein levels of LC3 and p62 as well as AR were determined by Western blotting using GAPDH as a loading control. **B**, AR negative DU145 cells were transfected with pEGFP-C1-AR (EGFP-AR) or empty vector (EGFP-V) in the presence of miR-101 inhibitor (miR-101 Inh) or negative control (NC). Cells were incubated in celastrol (2.0 μM, CEL) for an additional 24 h. MiR-101 levels were determined by RT-PCR. #, *P*<0.05 *versus* EGFP-V plus NC transfections. *, *P*<0.05 between EGFP-V and EGFP-AR in the presence of miR-101 inhibitor. The protein levels of AR, LC3and p62 were detected by Western blotting. **C**, DU145 cells were transfected with pGL3-B-miR-101-W (with wild type AR binding site) or pGL3-B-miR-101-M (with mutant AR binding site), along with AR expression vector (EGFP-AR) or empty vector (EGFP-V) for 24 h, then treated with DMSO or celastrol (CEL, 2.0 μM) for additional 24 h. MiR-101 levels were determined by RT-PCR. #, *P*<0.05 *versus* EGFP-V plus pGL3-B-miR-101-M transfections. *, *P*<0.05 between pGL3-B-miR-101-M and pGL3-B-miR-101-W transfections. The protein levels of AR, LC3 and p62 were detected by Western blotting using GAPDH as a loading control.

Next, we determined if AR negatively regulates celastrol-induced autophagy through inhibition of miR-101 expression in prostate cancer cells. MiR-101 mimic was used to increase the miR-101 level in LNCaP cells. With miR-101 upregulation by addition of miR-101 mimic (Fig 5A, upper), basal level autophagy was inhibited (LC3-II/LC3-I ratio decreased by half) (Fig 5A, bottom). Although AR reduction by celastrol was favorable for autophagy induction, addition of miR-101 mimic resulted in autophagy inhibition as shown by the half-fold decrease of LC3-II/LC3-I ratio and increase of p62 level (Fig 5A, bottom). In DU145 cells, stable expression of exogenous AR could upregulate miR-101 expression and could suppress autophagy triggered by celastrol. When miR-101 was inhibited by addition of miR-101 inhibitor, as determined by qPCR (Fig 5B, upper), autophagy was rescued regardless of AR overexpression (Fig 5B, bottom). These data suggest that the negative role AR played in the regulation of autophagy depends on its downstream targets. Furthermore, a pair of miR-101 expression constructs, pGL3-B-miR-101-W that contains the wild type ARE, and pGL3-B-miR-101-M that has mutant ARE were generated. pGL3-B-miR-101-W significantly enhanced miR-101 expression than pGL3-B-miR-101-M in co-transfection with exogenous AR in DU145 cells with or without celastrol treatment (Fig 5C). In accordance with the miR-101 levels, co-transfection with pGL3-B-miR-101-W that can be transactivated by AR showed more suppression on autophagy at the basal level or celastrol induced compared with pGL3-B-miR-101-M that could not be recognized by AR (Fig 5C), indicating that AR inhibits celastrol-induced autophagy via transactivation of miR-101.

### AR modulates miR-101 expression without affecting cell death

**Fig 6:**
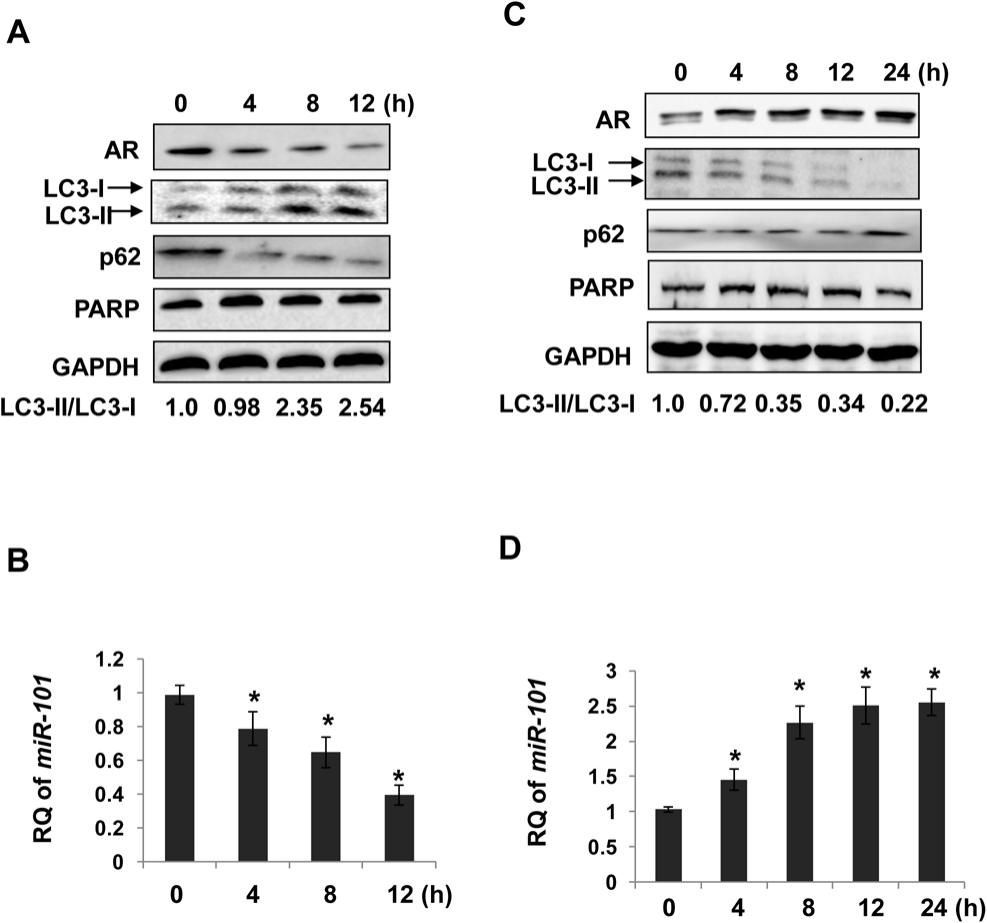
AR modulates miR-101 levels without affecting cell death. **A** and **B**, LNCaP cells were treated with MDV3100 at 5 μM for indicated times. Protein extracts were immunoblotted with antibodies against PARP, AR, LC3, p62 and GAPDH (loading control) (**A**). Mature miR-101 levels were determined by qPCR (**B**). **C** and **D**, LNCaP cells were subjected to androgen starvation as described in "**Materials and Methods**", followed by 1 nM of R1881 treatment for indicated times. PARP, AR, LC3, p62 and GAPDH (loading control) were detected by Western blotting (**C**). Mature miR-101 levels were determined by qPCR (**D**). Asterisks denote significance compared with control (0 h). *, *P* <0.05.

Since AR reduction also results in reduced cell viability, whether the observed miR-101 suppression and autophagy induction were due to cell death was determined. LNCaP cells were treated with AR antagonist MDV3100 (Enzalutamide) for different times. As expected, AR protein was decreased (Fig 6A). Along with AR reduction, miR-101 expression was decreased while autophagy was induced, as shown by decreased p62 levels and increased LC3-II/LC3-I ratios. In the same treated samples, PARP was kept intact (Fig 6A and 6B), indicating that cell viability was not affected post MDV3100 treatment at current conditions. Reversely, AR was activated by R1881 (Fig 6C). With AR activation, miR-101 expression was increased (Fig 6D) along with autophagy inhibition (Fig 6C and 6D). Again, cell death was not observed post R1881 treatment (Fig 6C). These results indicate that miR-101 reduction was due to AR suppression, but not cell death.

### Autophagy inhibition with miR-101 potentiates the cytotoxic effects of celastrol

**Fig 7:**
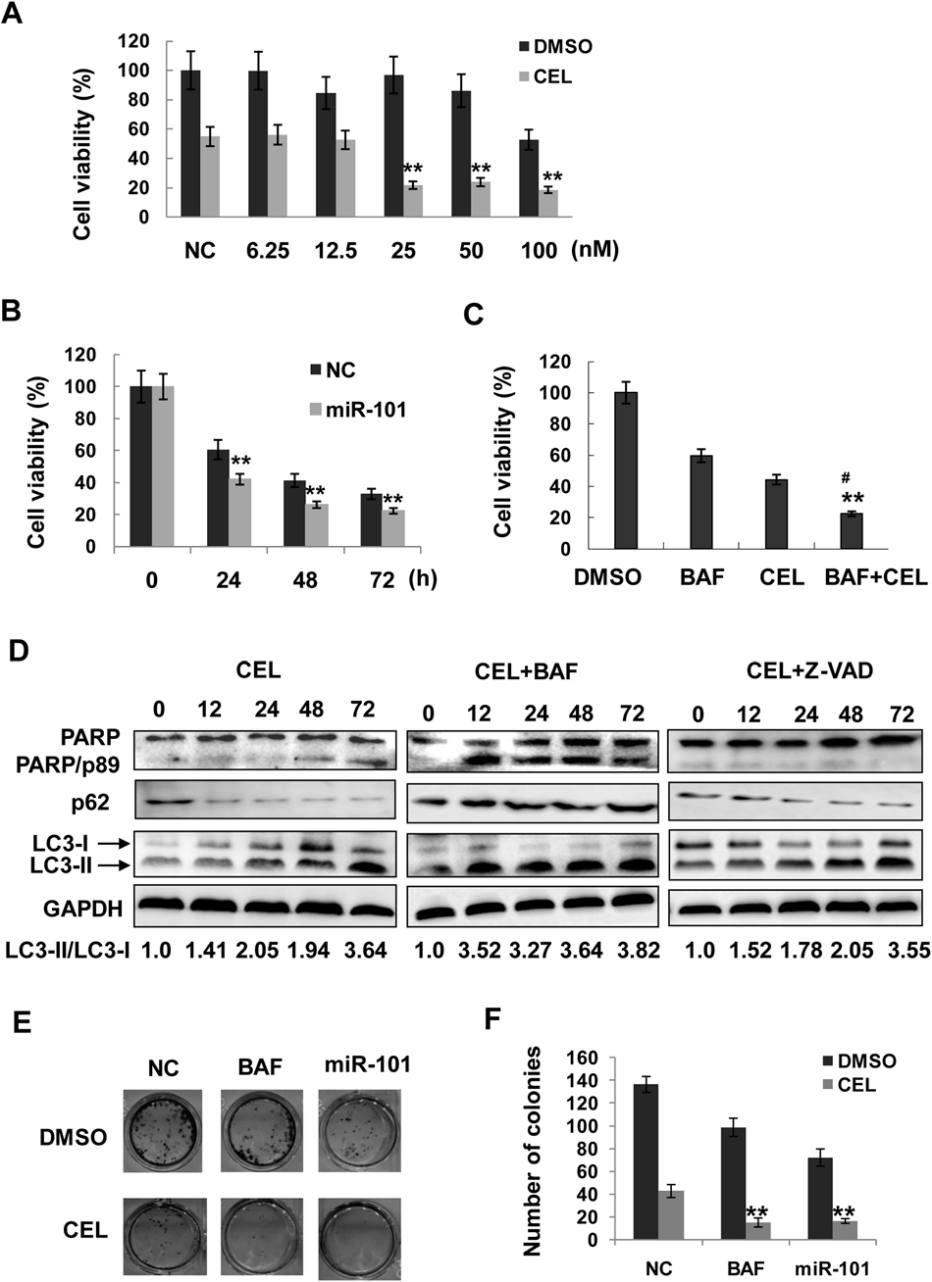
Autophagy potentiates the effects of celastrol on inhibition of cell proliferation and induction of apoptosis. Cell viability was measured by MTT assay after treatment with celastrol. **A**, LNCaP cells were transfected with negative control (NC) or miR-101 mimic at indicated concentrations for 24 h, followed by additional 96 h treatment with DMSO or celastrol at 1.5 μM. **, *P*<0.01 *versus* NC with celastrol treatment. **B**, LNCaP cells were treated with celastrol (1.5 μM) in the presence of miR-101 mimic or negative control (NC) for indicated time points. **, *P*<0.01 *versus* NC. **C**, LNCaP cells were treated with celastrol (CEL, 1.5 μM) or DMSO in the presence or absence of bafilomycin A1 (BAF, 10 nM) for 24 h. #, *P* <0.05 *versus* CEL, **, *P*<0.01 *versus* other two groups. **D**, LNCaP cells were treated with celastrol (CEL, 2.0 μM) in the presence or absence of bafilomycin A1 (BAF, 10 nM) or Z-VAD (20 μM) for up to 72 h. Protein extracts were immunoblotted with antibodies against PARP, LC3, p62 and GAPDH (loading control). **E**, Colony formation assay. LNCaP cells were treated with DMSO or celastrol (CEL, 1.5 μM) in the presence of miR-101 mimic or negative control (NC) or bafilomycin A1 (BAF, 10 nM) for 24 h. Colony formation was performed as described in the **Materials and Methods**. Colonies with over 50 cells were counted (**E**) and quantified in **F.** **, *P*<0.01 *versus* NC with celastrol treatment.

Autophagy plays dual roles in cancer cell survival under stresses, either protects cancer cells from apoptosis or itself leads to cell death, termed as type II programmed cell death. To evaluate the effect of autophagy on cell growth upon celastrol treatment, miR-101 mimic was used to inhibit autophagy and cell proliferation was determined post celastrol treatment. As shown in Fig 7, cell viability after celastrol treatment was significantly decreased by miR-101 mimic compared with the negative control (Fig 7A and 7B). Similarly, treatment with autophagy inhibitor bafilomycin A1 further decreased cell viability in comparison with celastrol treatment alone (Fig 7C). Prolonged treatment (up to 72 h) with celastrol induced apoptosis in LNCaP cells as shown by PARP fragment (p89 kD), a product of caspase-3 cleavage (Fig 7D). When autophagy was blocked with the inhibitor of autophagic flux Bafilomycin A1, the onset of celastrol-induced cell death was shifted to earlier time points. When apoptosis was blocked with the pan-inhibitor of caspase, celastrol-induced autophagy remained largely unaltered (Fig 7D). These results demonstrate that autophagy can delay the onset of apoptosis induced by celastrol in the investigated cell line. To see long term effect of autophagy on cell growth, colony formation was performed. As shown in Fig 7E and 7F, inhibition of autophagy by miR-101 or bafilomycin A1 enhanced celastrol-induced reduction on colonies formation. These results indicate that autophagy serves as a cytoprotective mechanism for prostate cancer cell survival upon celastrol treatment.

## Discussion

AR plays a critical role in the growth of prostate cancer cells, thus targeting AR signaling pathway is an effective strategy for prostate cancer treatment. Celastrol has been reported targeting AR signaling by promoting AR degradation through inhibition of Hsp90 or activation of calpain [5, 6]. AR reduction by celastrol in prostate cancer xonegraft tissues demonstrates its potential application for prostate cancer treatment [3, 18]. Suppression on AR axis has been proved to be associated with autophagy regulation. Blocking AR signaling pathway by using anti-androgen bicalutamide or knocking down AR in AR-positive PCa cell lines resulted in increased autophagy [8, 11]. Transfection of functional AR in AR-negative PCa cell lines led to a decreased autophagy [10]. In addition, AR is mutated in LNCaP cells, allowing the cells to respond to a variety of stimuli [19]. AR activation by non-androgens could also inhibit autophagy [20], further confirming that AR may play a negative role in the regulation of autophagy. Consistent with these findings, our present study showed that celastrol induced autophagy in AR-positive prostate cancer cells, which was correlated with AR protein reduction. Ectopic expression of AR in AR-negative prostate cancer cells, or gain of function of the AR signaling in AR-positive cells, led to suppression of autophagy, demonstrating the negative role of AR in celastrol-induced autophagy in prostate cancer cells.

The exact mechanism underlying AR inhibition and autophagy induction has not been fully understood. AMPK activation or Grp78 downregulation has been proposed as the underlying mechanisms for autophagy induction upon androgen deprivation [8, 9]. Prolonged AR suppression with Enzalutamide was accompanied by AMPK activation, which eventually led to inhibition of autophagy inhibitor mTOR [9]. However, as a transcription factor, how AR regulates autophagy associated molecules at the transcriptional level remains unclear. MiR-101 is an intracellular inhibitor of autophagy [12]. Here, we showed that AR inhibited autophagy through transactivation of the expression of miR-101. A typical AR binding site was verified in the upstream region of *miR-101*gene by luciferase reporter and ChIP assays. MiR-101 expression correlated with AR status in prostate cancer cell lines. Celastrol-induced autophagy was suppressed by AR, but compromised by using miR-101 inhibitor. Transfection of miR-101 led to inhibition of celastrol-induced autophagy in spite of AR depletion. Furthermore, mutagenesis of the AR binding site in *miR-101* gene led to decreased suppression of autophagy by AR.

Our work demonstrates the negative regulation of AR/miR-101 on celastrol-induced autophagy in AR positive prostate cancer cells. We found that celastrol could also induce autophagy in AR negative cells, which could be suppressed by exogenous AR. These results support the negative regulation of AR on celastrol-induced autophagy that was found in AR positive cells. It has been reported that celastrol induces autophagy in AR negative prostate cancer cell line such as PC-3 as well as other type of cancers such as pancreatic cancer and hepatocarcinoma [21, 22]. Activation of HIF-1/BNIP3 has been proved to be a mechanism for celastrol to induce autophagy in those types of cells [22]. Exogenous AR could increase miR-101 expression and miR-101 mimic could suppress celastrol-induced autophagy in AR negative cells, demonstrating the negative regulation of exogenous AR/miR-101 on autophagy induction upon celastrol treatment in these cells. miR-101 is also expressed in AR negative prostate cancer cells, but at lower levels compared to AR positive cells. Thus, *miR-101* transactivation depends on other transcriptional factors than AR, as EZH is reported to be one such factor in these cells [13].

Abiraterone and Enzalutamide are representatives of the second generation of androgen deprivation therapy (ADT) that have been moved into clinical trials for the treatment of metastatic prostate cancer [23–25]. Androgen deprivation by Enzalutamide or Bicalutamide induced autophagy in LNCaP and C4-2B cells, and conferred resistance to apoptosis [26, 27]. Targeting AR with AR degrader ASC-J9 led to prostate cancer suppression via the induction of autophagy in CWR22R cells [10]. In the current study, we found that autophagy inhibition by inhibitor miR-101 or bafilomycin A1 could enhance LNCaP prostate cancer cell response to celastrol treatment. Since AR signaling inhibition triggered autophagy may cause pro-survival or anti-survival response depending on specific cell context as well as cell-drug interaction, our results suggest that autophagy inhibition should take into account for celastrol future application.

In summary, our results demonstrate that AR plays a negative role of in the regulation of celastrol-induced autophagy; it suppresses autophagy via transactivation of *miR-101* in prostate cancer cells. Targeting AR with celastrol in the presence of miR-101 would be in favor of the inhibition on cell proliferation in prostate cancer cells.

## References

[1] Mehrpour M, Esclatine A, Beau I, Codogno P. Overview of macroautophagy regulation in mammalian cells. Cell Res. 2010; 20(7): 748–62. 10.1038/cr.2010.82 20548331

[2] Yang Z, Klionsky DJ. An overview of the molecular mechanism of autophagy. Curr Top Microbiol Immunol. 2009; 335: 1–32. 10.1007/978-3-642-00302-8_1 19802558 PMC2832191

[3] Shao L, Zhou Z, Cai Y, Castro P, Dakhov O, Shi P, et al Celastrol suppresses tumor cell growth through targeting an AR-ERG-NF-kappaB pathway in TMPRSS2/ERG fusion gene expressing prostate cancer. PLoS ONE. 2013; 8(3): e58391 10.1371/journal.pone.0058391 23554889 PMC3590152

[4] Yang H, Chen D, Cui QC, Yuan X, Dou QP. Celastrol, a triterpene extracted from the Chinese "Thunder of God Vine," is a potent proteasome inhibitor and suppresses human prostate cancer growth in nude mice. Cancer Res. 2006; 66(9): 4758–65. 16651429 10.1158/0008-5472.CAN-05-4529

[5] Hieronymus H, Lamb J, Ross KN, Peng XP, Clement C, Rodina A, et al Gene expression signature-based chemical genomic prediction identifies a novel class of HSP90 pathway modulators. Cancer Cell. 2006; 10(4): 321–30. 17010675 10.1016/j.ccr.2006.09.005

[6] Yang H, Murthy S, Sarkar FH, Sheng S, Reddy GP, Dou QP. Calpain-mediated androgen receptor breakdown in apoptotic prostate cancer cells. J Cell Physiol. 2008; 217(3): 569–76. 10.1002/jcp.21565 18726991 PMC2597227

[7] Reddy GP, Barrack ER, Dou QP, Menon M, Pelley R, Sarkar FH, et al Regulatory processes affecting androgen receptor expression, stability, and function: potential targets to treat hormone-refractory prostate cancer. J Cell Biochem. 2006; 98(6): 1408–23. 16619263 10.1002/jcb.20927

[8] Bennett HL, Fleming JT, O'Prey J, Ryan KM, Leung HY. Androgens modulate autophagy and cell death via regulation of the endoplasmic reticulum chaperone glucose-regulated protein 78/BiP in prostate cancer cells. Cell Death Dis. 2010; 1: e72 10.1038/cddis.2010.50 21364676 PMC3032338

[9] Chhipa RR, Wu Y, Ip C. AMPK-mediated autophagy is a survival mechanism in androgen-dependent prostate cancer cells subjected to androgen deprivation and hypoxia. Cell Signal. 2011; 23(9): 1466–72. 10.1016/j.cellsig.2011.04.008 21554950 PMC3115439

[10] Jiang Q, Yeh S, Wang X, Xu D, Zhang Q, Wen X, et al Targeting androgen receptor leads to suppression of prostate cancer via induction of autophagy. J Urol. 2012; 188(4): 1361–8. 10.1016/j.juro.2012.06.004 22906664

[11] Li M, Jiang X, Liu D, Na Y, Gao GF, Xi Z. Autophagy protects LNCaP cells under androgen deprivation conditions. Autophagy. 2008; 4(1): 54–60. 17993778 10.4161/auto.5209

[12] Frankel LB, Wen J, Lees M, Hoyer-Hansen M, Farkas T, Krogh A, et al microRNA-101 is a potent inhibitor of autophagy. EMBO J. 2011; 30(22): 4628–41. 10.1038/emboj.2011.331 21915098 PMC3243595

[13] Cao P, Deng Z, Wan M, Huang W, Cramer SD, Xu J, et al MicroRNA-101 negatively regulates Ezh2 and its expression is modulated by androgen receptor and HIF-1alpha/HIF-1beta. Mol Cancer. 2010; 9: 108 10.1186/1476-4598-9-108 20478051 PMC2881117

[14] Shi R, Chiang VL. Facile means for quantifying microRNA expression by real-time PCR. Biotechniques. 2005; 39(4): 519–25. 16235564 10.2144/000112010

[15] Frezza M, Yang H, Dou QP. Modulation of the tumor cell death pathway by androgen receptor in response to cytotoxic stimuli. J Cell Physiol. 2011; 226(11): 2731–9. 10.1002/jcp.22758 21448923 PMC3134581

[16] Yang H, Wu GS. p53 Transactivates the phosphatase MKP1 through both intronic and exonic p53 responsive elements. Cancer Biol Ther. 2004; 3(12): 1277–82. 15611668 10.4161/cbt.3.12.1370

[17] Lippai M, Low P. The role of the selective adaptor p62 and ubiquitin-like proteins in autophagy. Biomed Res Int. 2014; 2014: 832704 10.1155/2014/832704 25013806 PMC4075091

[18] Huang W, He T, Chai C, Yang Y, Zheng Y, Zhou P, et al Triptolide inhibits the proliferation of prostate cancer cells and down-regulates SUMO-specific protease 1 expression. PLoS ONE. 2012; 7(5): e37693 10.1371/journal.pone.0037693 22666381 PMC3364364

[19] Castoria G, Giovannelli P, Di Donato M, Ciociola A, Hayashi R, Bernal F, et al Role of non-genomic androgen signalling in suppressing proliferation of fibroblasts and fibrosarcoma cells. Cell Death Dis. 2014; 5: e1548 10.1038/cddis.2014.497 25476896 PMC4649827

[20] Kung HJ. Targeting tyrosine kinases and autophagy in prostate cancer. Horm Cancer. 2011; 2(1): 38–46. 10.1007/s12672-010-0053-3 21350583 PMC3020299

[21] Zhao X, Gao S, Ren H, Huang H, Ji W, Hao J. Inhibition of autophagy strengthens celastrol-induced apoptosis in human pancreatic cancer in vitro and in vivo models. Curr Mol Med. 2014;14(4):555–63. 24730520 10.2174/1566524014666140414211223

[22] Han X, Sun S, Zhao M, Cheng X, Chen G, Lin S, et al Celastrol stimulates hypoxia-inducible factor-1 activity in tumor cells by initiating the ROS/Akt/p70S6K signaling pathway and enhancing hypoxia-inducible factor-1α protein synthesis. PLoS One. 2014; 9(11):e112470 10.1371/journal.pone.0112470 25383959 PMC4226555

[23] Beer TM, Armstrong AJ, Rathkopf DE, Loriot Y, Sternberg CN, Higano CS, et al Enzalutamide in metastatic prostate cancer before chemotherapy. N Engl J Med. 2014; 371(5): 424–33. 10.1056/NEJMoa1405095 24881730 PMC4418931

[24] Hoy SM. Abiraterone acetate: a review of its use in patients with metastatic castration-resistant prostate cancer. Drugs. 2013; 73(18): 2077–91. 10.1007/s40265-013-0150-z 24271422

[25] Tombal B, Borre M, Rathenborg P, Werbrouck P, Van Poppel H, Heidenreich A, et al Enzalutamide monotherapy in hormone-naive prostate cancer: primary analysis of an open-label, single-arm, phase 2 study. Lancet Oncol. 2014; 15(6): 592–600. 10.1016/S1470-2045(14)70129-9 24739897

[26] Boutin B, Tajeddine N, Vandersmissen P, Zanou N, Van Schoor M, Mondin L, et al Androgen deprivation and androgen receptor competition by bicalutamide induce autophagy of hormone-resistant prostate cancer cells and confer resistance to apoptosis. Prostate. 2013; 73(10): 1090–102. 10.1002/pros.22658 23532738

[27] Nguyen HG, Yang JC, Kung HJ, Shi XB, Tilki D, Lara PN, et al Targeting autophagy overcomes Enzalutamide resistance in castration-resistant prostate cancer cells and improves therapeutic response in a xenograft model. Oncogene. 2014; 33(36): 4521–30. 10.1038/onc.2014.25 24662833 PMC4155805

